# Sanguinarine Induces Apoptosis Pathway in Multiple Myeloma Cell Lines via Inhibition of the JaK2/STAT3 Signaling

**DOI:** 10.3389/fonc.2019.00285

**Published:** 2019-04-17

**Authors:** Sabah Akhtar, Iman W. Achkar, Kodappully S. Siveen, Shilpa Kuttikrishnan, Kirti S. Prabhu, Abdul Q. Khan, Eiman I. Ahmed, Fairooz Sahir, Jayakumar Jerobin, Afsheen Raza, Maysaloun Merhi, Hesham M. Elsabah, Ruba Taha, Halima El Omri, Hatem Zayed, Said Dermime, Martin Steinhoff, Shahab Uddin

**Affiliations:** ^1^Translational Research Institute, Academic Health System, Hamad Medical Corporation, Doha, Qatar; ^2^Translational Cancer Research Facility, Translational Research Institute, Hamad Medical Corporation, Doha, Qatar; ^3^National Center for Cancer Care and Research, Hamad Medical Corporation, Doha, Qatar; ^4^Department of Biomedical Sciences, College of Health Sciences, Qatar University, Doha, Qatar; ^5^Department of Dermatology Venereology, Hamad Medical Corporation, Doha, Qatar; ^6^Weill Cornell-Medicine, Doha, Qatar; ^7^Weill Cornell-Medicine, Cornell University, New York, NY, United States

**Keywords:** apoptosis, caspases, STAT3, sanguinarine, multiple myeloma, hematological malignancy

## Abstract

Sanguinarine (SNG), a benzophenanthridine alkaloid, has displayed various anticancer abilities in several vivo and *in vitro* studies. However, the anticancer potential of SNG is yet to be established in multiple myeloma (MM), a mostly incurable malignancy of plasma cells. In this study, we aimed to investigate the potential anti-proliferative and pro-apoptotic activities of SNG in a panel of MM cell lines (U266, IM9, MM1S, and RPMI-8226). SNG treatment of MM cells resulted in a dose-dependent decrease in cell viability through mitochondrial membrane potential loss and activation of caspase 3, 9, and cleavage of PARP. Pre-treatment of MM cells with a universal caspase inhibitor, Z-VAD-FMK, prevented SNG mediated loss of cell viability, apoptosis, and caspase activation, confirming that SNG-mediated apoptosis is caspase-dependent. The SNG-mediated apoptosis appears to be resulted from suppression of the constitutively active STAT3 with a concomitant increase in expression of protein tyrosine phosphatase (SHP-1). SNG treatment of MM cells leads to down-regulation of the anti-apoptotic proteins including cyclin D, Bcl-2, Bclxl, and XIAP. In addition, it also upregulates pro-apoptotic protein, Bax. SNG mediated cellular DNA damage in MM cell lines by induction of oxidative stress through the generation of reactive oxygen species and depletion of glutathione. Finally, the subtoxic concentration of SNG enhanced the cytotoxic effects of anticancer drugs bortezomib (BTZ) by suppressing the viability of MM cells via induction of caspase-mediated apoptosis. Altogether our findings demonstrate that SNG induces mitochondrial and caspase-dependent apoptosis, generates oxidative stress, and suppresses MM cell lines proliferation. In addition, co-treatment of MM cell lines with sub-toxic doses of SNG and BTZ potentiated the cytotoxic activity. These results would suggest that SNG could be developed into therapeutic agent either alone or in combination with other anticancer drugs in MM.

## Introduction

Multiple myeloma (MM) is a hematological malignancy characterized by the proliferation of clonal plasma cells in the bone marrow (BM) accompanied by secretion of monoclonal immunoglobulin (1). As standard chemotherapy and hematopoietic stem-cell transplantation still do not offer a cure for patients, alternative strategies are required for therapeutic intervention (1).

Signal transducer and activator of transcription 3 (STAT3) is a member of the STAT family of transcription factors as well as a critical molecule of JAK/STAT signaling pathway (2, 3). Deregulated STAT3 is oncogenic and has been shown to be involved in the regulation of various important functions such as cell proliferation, and differentiation in many cancer cells. Constitutive and sustained activation of STAT3 has been observed in many human malignancies including multiple myeloma, leukemia, lymphoma, and solid tumors (4, 5). Activation of STAT3 has been found to be associated with shorter survival of patients with multiple myeloma (6). A number of pro-survival and antiapoptotic genes are regulated by activation of STAT3 at the transcription level including cyclin D, Bclxl, Bcl2, survivin, MMP9, and VEGF (7, 8). Protein tyrosine phosphatases (PTPs) are negative regulators of the JAK/STAT signaling. SHP-1 is one of PTP members that has been shown to dephosphorylate JAK kinases (9) and STAT3 directly (10) to prevent the JAK/STAT pathway.

Sanguinarine (SNG) a benzophenanthridine alkaloid, said to be a “secondary metabolite” or “natural product” (11, 12) is predominantly isolated from the root of *Sanguinaria canadensis* (13). *In vivo* and *in vitro* preliminary pre-clinical studies in animal models have reported SNG anticancer potential via the induction of apoptosis and/or anti-proliferative, anti-angiogenic, and anti-invasive activity which has been well-documented in a wide range of cancers (14–16) including lung (17–21), breast (22–28) skin cancers (12, 29–32), and hematological malignancies (33–38). Interestingly, SNG does not show toxicity in healthy cells signifying its potential for anticancer agents (39). SNG has been shown to induce cell death via the extrinsic and intrinsic apoptotic pathways (14). Inhibition of more than 70% of tumor growth has been seen via SNG-mediated production of reactive oxygen species (ROS), inducing oxidative stress and cell damage in cancer cells (16). In addition, SNG exhibits cytotoxic effects via suppressing the activity of various signaling cascades in a wide range of cancer cell lines (15, 31, 32, 40, 41). Although the anticancer activity of SNG has been shown in hematological malignancies, mainly leukemias and lymphomas but its anticancer potential has not been studied in multiple myeloma.

In this study, we investigated the anticancer activity of SNG in MM cell lines. Our data showed that SNG treatment of MM cells suppressed the viability via induction of apoptosis. SNG treatment of MM cells inactivated STAT3 activity with concomitant upregulation of SHP-1, a PTPs that is a negative regulator of STAT3. Furthermore, SNG-induced apoptosis involves mitochondrial and caspase-cascade signaling pathway. SNG mediated apoptosis was found to involve ROS due to depletion of glutathione in MM cells. In addition, SNG potentiated the anticancer effects of bortezomib in MM cell lines.

## Materials and Methods

### Reagents and Antibodies

Sanguinarine chloride, Cell Counting Kit-8 (CCK-8), and N-acetylcysteine (NAC) were purchased from Sigma Chemical Co. (St. Louis, USA). Z-VAD-FMK was purchased from Calbiochem (San Diego, USA). Antibodies against caspase-9, Bclxl, Bcl2, phospho-STAT3, STAT3, SHP-1, cleaved caspase-3, and caspase-3 were purchased from Cell Signaling Technologies (Beverly, USA). VELCADE® (Bortezomib), PARP, and GAPDH antibodies were purchased from Santa Cruz Biotechnology, Inc. (Santa Cruz, USA). XIAP antibody was purchased from Abcam (Cambridge, UK). FITC Annexin V apoptosis detection kit I, Apo-Direct kit, Fixation/Permeabilization solution kit, BD MitoScreen (JC-1), BV421 mouse anti-γH2AX (pS139), PE rabbit anti-active caspase-3, and Alexa Fluor 700 mouse anti-cleaved PARP (Asp214) antibodies were purchased from BD Biosciences (San Jose, USA). CellROXGreen and ThiolTracker Violet were purchased from Invitrogen (Massachusetts, USA). RPMI 1640, fetal bovine serum (FBS), Penicillin Streptomycin (PenStrep) were purchased from Life technologies (California, USA).

### Cell Culture

U266, MM1S, IM9, and RPMI-8226 cells were obtained from ATCC, USA, and grown in RPMI 1,640 medium supplemented with 10% (v/v) fetal bovine serum and 100 U/ml of Pen Strep at 37°C in a humidified incubator with 5% CO_2_.

### Cell Viability Assays

Briefly, 1 × 10^4^ cells grown in 96-well cell culture plates (0.2 mL media) were treated with increasing doses of SNG. After the incubation period (24 h), 10 μL of CCK-8 reagent was added to the wells, followed by 2 h incubation at 37°C. Finally, the optical density was measured at 450 nm and percent cell viability was calculated as described previously (42).

### AnnexinV/propidium Iodide Dual Staining

U266, MM1S, and IM9 cell lines were treated with various doses of SNG for 24 h. The cells were stained with annexin V-FITC and propidium iodide as described earlier (42), and then analyzed by flow cytometry.

### Cell Lysis and Immunoblotting

SNG treated U266, MM1S, IM9, and RPMI cells were lysed with Laemmli buffer as described previously (43), an equal quantity of protein was separated by SDS-PAGE, transferred to PVDF membrane, and then immunoblotted using various antibodies and then visualized.

### TUNEL Assay for Measurement of DNA Double-Strand Breaks

DNA fragmentation in the MM cells was measured using Apo-Direct kit as described earlier (44). Briefly, SNG treated cells were initially fixed with 1% paraformaldehyde for 30 min, permeabilized using ice-cold 70% ethanol, and then incubated with 50 μL DNA labeling solution for 60 min at 37°C. Stained cells were washed and then analyzed using LSRFortessa (Beckon Dickinson, San Diego, USA).

### Colony Formation Assay

Effect of SNG on the anchorage-independent growth of MM cells was assayed by colony formation assay following the CytoSelect 96-well cell transformation assay kit's protocol provided by the manufacturer (Cell Biolabs, Inc.) Briefly, U266 and MM1S cell lines were treated with SNG (0.25, 0.5, 1, 2, and 4 μM) and seeded in a density of 4 × 10^5^ cell/ml into a 0.4% top agar layer poured over 0.6% lower agar layer and incubated for 8 days at 37°C and 5% CO_2_. Then agar was solubilized, and cells were lysed. Following that, the CyQuant dye was used to measure the fluorescence intensity of each well at 485/530 nm.

### Comet Assay

The DNA damage in multiple myeloma cells was evaluated using comet SCGE assay kit obtained from Enzo life sciences. The multiple myeloma cells 1 × 10^5^/ml at a ratio of 1:10 were mixed with low melting agarose, and 75 μl of the mixture was added to comet slide. The slides were kept in the dark for 10 min and then immersed in lysis solution followed by alkaline solution and finally 1X TBE buffer. The slides were then transferred to a horizontal gel electrophoresis apparatus, and power was supplied at 1 volt per cm. Once electrophoresis was completed the slides were dipped in 70% ethanol and air dried. Then 100 μl of diluted CYGREEN® nucleic acid dye was added to each circle of comet slide and stained for 30 min in the dark. The slides were dried completely and visualized using epifluorescence microscopy.

### Measurement of Active Caspase-3 and Cleaved PARP by Flow Cytometry

U266, MM1S, and IM9 cells were treated with increasing concentrations of SNG as described in the figure legend. The cells were fixed and permeabilized using Fixation/Permeabilization solution kit, according to the manufacturer's protocol. 0.5 × 10^6^ cells in 1x Perm-Wash buffer were stained with BV605 tagged anti-active caspase-3 and AF700 tagged PARP cleaved form antibodies (5.0 μl each) for 30 min, washed twice and then analyzed by flow cytometry using BD LSRFortessa analyzer as described previously (42).

### Measurement of Bax/Bcl2 by Flow Cytometry

U266 cells were treated with SNG, fixed, and permeabilized using Fixation/Permeabilization solution kit, according to the manufacturer's protocol. 0.5 × 10^6^ cells in 1x Perm-Wash buffer were stained with FITC tagged anti-bax and V450 tagged Bcl-2 antibodies (5.0 μl each) for 30 min, washed twice and then analyzed by flow cytometry. Matched isotype control was prepared for all treatment conditions. The Bax/Bcl-2 ratio was determined by dividing the relative median fluorescence intensity (MFI) of Bax staining by the relative MFI of Bcl-2 staining. Relative MFI was determined by subtracting the MFI of the staining isotype control from the MFI of the monoclonal antibody.

### Measurement of Mitochondrial Membrane Potential (MMP)

Effect of SNG on the MMP of MM cells was determined using BD MitoScreen (JC-1) assay kit as described earlier (45). After incubation with SNG, cells in 1x assay buffer were incubated with JC-1 stain for 15 min at 37°C, washed and then analyzed by Flow Cytometry, to quantify the reduction in red fluorescence.

### Cytochrome C Release Assay

U266 cells were treated with SNG (1, 2, and 4 μM) for 24 h and then cytosolic as well as mitochondrial fractions were isolated as described previously (46). Briefly, cells were harvested, incubated in ice-cold hypotonic buffer and then homogenized by passing through a 22-gauge needle (15–20 times). Mitochondrial pellet and cytosolic fraction were obtained by differential centrifugation. Twenty microgram protein from the cytosolic and mitochondrial fractions was separated by SDS-PAGE and then immunoblotted using anti-cytochrome c antibody.

#### Gene Silencing of STAT3 Using Small Inference RNA Technique

U266 cells (1 × 10^6^) were transfected with STAT3 siRNA [Cat 4390824, Life Technologies, California, USA)] and *Control siRNA* (Cat no: *1027281*, Qiagen) using 4D-Nucleofector™ System (Lonza). After 48 h of incubation, cells were lysed and immunoblotted with anti-STAT3 and other antibodies.

### Measurement of Reactive Oxygen Species

MM cells, IM9, and U266, were exposed to various doses of SNG for a period of 24 h. After incubation, cells were harvested, washed, and then stained with CellROX Green reagent (5 μM in HBSS) for 30 min at 37°C. The stained cells were analyzed by flow cytometry as described earlier (42) to quantify the levels of reactive oxygen species.

### Measurement of Reduced Glutathione

MM cells IM9 and U266 were exposed to increasing doses of SNG for a period of 24 h. After incubation, cells were harvested, washed and then stained with ThiolTracker Violet reagent (5 μM in HBSS) for 30 min at 37°C. The stained cells were analyzed by flow cytometry as described earlier (42) to quantify the levels of reduced glutathione.

### Statistical Analysis

The significance of differences between different treatment groups was determined by one-way ANOVA with Sidak *post-hoc* test, performed using GraphPad Prism v7.0 (GraphPad Software Inc, California, USA). Values of *P* < 0.001 were considered statistically significant. ^*^ denotes *p* < 0.001. In all figures, data is expressed as the mean ± standard deviation (S.D), with the vertical error bars denoting the S.D.

## Results

### Treatment of MM Cells With SNG Mediates Cellular DNA Damage and Induces Apoptosis

**Figure 1:**
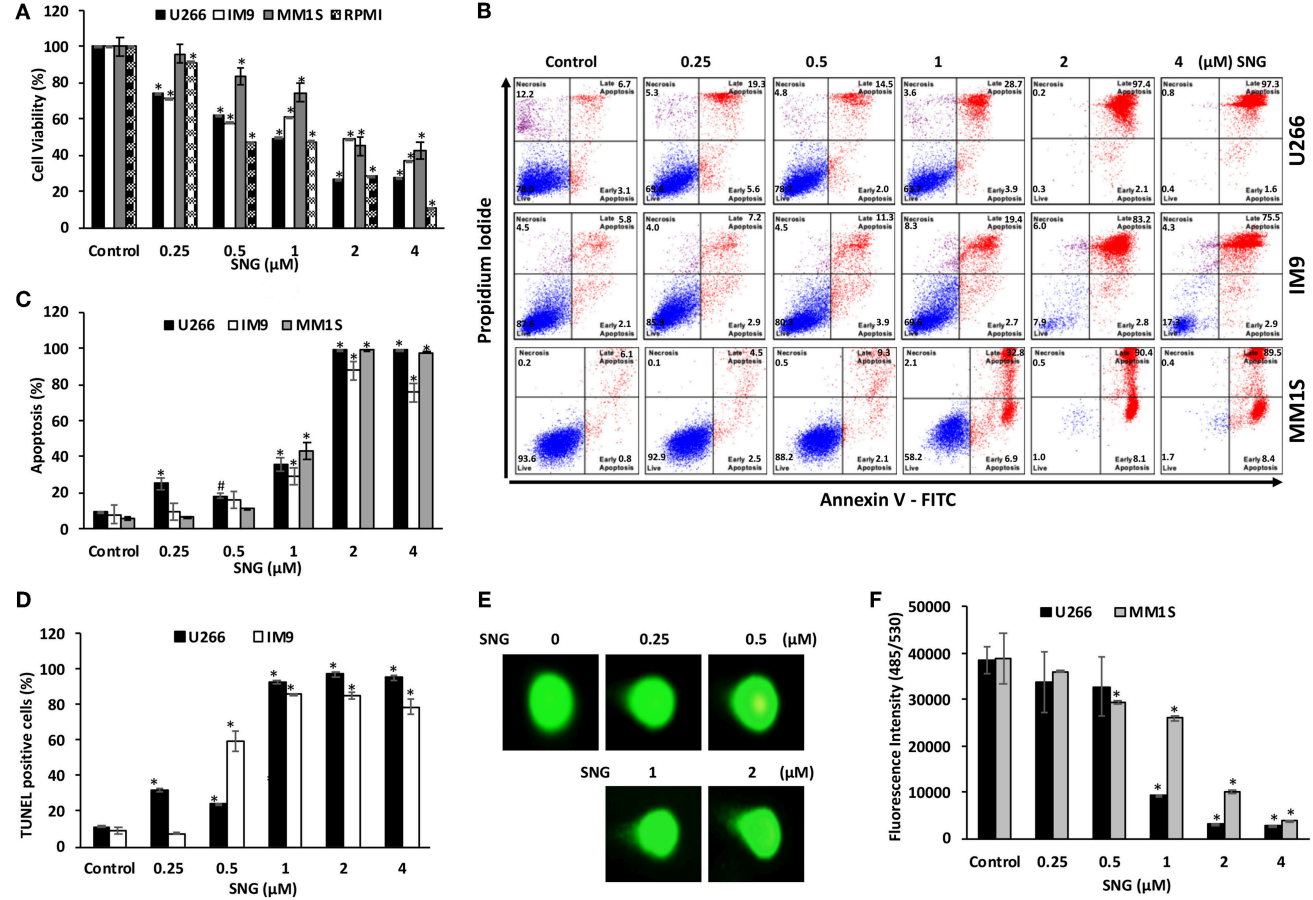
Effects of SNG on proliferation, and apoptosis in MM cells. **(A)** SNG inhibits the viability of MM cells. U266, IM9, MM1S, and RPMI cells were incubated with 0.25, 0.5, 1.0, 2.0, and 4 μM SNG for 24 h. Cell proliferation assays were performed using CCK-8 as described in Materials and Methods. The graph displays the mean ± SD (standard deviation) of three independent experiments with replicates of six wells for all the doses. *p < 0.001. **(B)** SNG induces apoptosis in MM cell lines. U266, IM-9, and MM1S cells were treated with 0.25, 0.5, 1.0, 2.0, and 4 μM of SNG for 24 h and cells were subsequently stained with fluorescein-conjugated annexin-V, PI, and subsequently analyzed by flow cytometry. **(C)** The graph displays the mean ± SD (standard deviation) of percentage of apoptotic cells (Early Apoptosis + Late Apoptosis) of two independent experiments with replicates of three wells for all the doses. ^#^*p* < 0.05, *p < 0.001. **(D)** SNG treatment induces DNA fragmentation in MM cells. U266 and IM9 cells were treated with and without 0.25, 0.5, 1.0, 2.0, and 4 μM of SNG as indicated for 24 h and cells were subsequently stained using the APO-DIRECT kit (BD Biosciences) to measure fragmentation using TUNEL assay (terminal deoxynucleotidyltransferase dUTP nick end labeling) as described in Materials and Methods and then analyzed by flow cytometry. The graph displays the mean ± SD (standard deviation) of three independent experiments for all the doses. *p < 0.001. **(E)** SNG treatment induces DNA damage in MM cells. IM-9 and U266 cells were treated with and without 0.25, 0.5, 1.0, 2, and 4.0 μM of SNG as indicated for 24 h and cells were used to perform Comet assay to visual DNA fragmentation as described in Materials and Methods and then analyzed by flow cytometry. **(F)** Antiproliferative effects of SNG on U266 and MM1S cells were analyzed by colony formation assay. U266 and MM1S cells were plated on methyl-cellulose with 0.25, 0.5, 1.0, 2, and 4.0 μM of SNG for 10 days. Colonies were stained with CyQuant dye and fluorescence intensity measured at 485/530 nm. The graph displays the mean ± SD (standard deviation) of three independent experiments for all the doses. *p < 0.001.

We first determined whether treatment of MM cells with SNG induces cellular damage. To achieve this objective, U266, MM1S, IM9, and RPMI-8226 cells were treated with an increasing dose of SNG for 24 h and viability was determined by cell viability assay using CCK-8 solution. As shown in Figure 1A, SNG treatment of U266 and RPMI-8226 cells significantly decreased MM cell viability in a dose-dependent manner when treated with 0.25–4 μM SNG, with the half maximal inhibitory concentration (IC_50_) between 1 and 2 μM. In the next series of experiments, we determined whether inhibition of cell viability is due to apoptosis, measurement of apoptosis was performed using various assays, including annexin V-FITC/PI dual staining and measurement of DNA fragmentation by TUNEL assay. Annexin V-FITC/PI dual staining showed that U266 treated with 0.25, 0.5, 1, 2, and 4 μM SNG had a dose-dependent increase in apoptosis (Figure 1B). A similar pattern of apoptotic response was observed in IM9 and MM1S cell lines (Figures 1B,C). DNA ds breaks were measured by means of a TUNEL assay, using the fragmentation marker dUTP. This was carried out on U266 cells treated with 0.25, 0.5, 1, 2, and 4 μM SNG, which revealed 11% control cells containing DNA ds breaks with over 92% of cells containing ds breaks when treated with 1–4 μM SNG (Figure 1D). DNA ds breaks were further confirmed by measuring the staining with p-H2X, a marker for DNA ds breaks (Supplementary Figure 1A). In addition, SNG induced cellular DNA damage in individual cells could be visually deduced by a COMET assay which revealed significant DNA damage in a dose-dependent manner (Figure 1E). Finally, we assessed the effect of SNG on colony formation ability of MM cells. U266 and MM1S cell were treated with various doses of SNG and seeded on agar using CytoSelect; Cell Biolabs assay kit. After 8 days of incubation, colonies were quantified as described in material methods. Using a fluorescence plate reader (485/520 nm filter set) according to the assay protocol. As shown in Figure 1F and Supplementary Figures 1B,C, SNG treatment causes a dose-dependent decrease in colony formation ability in both cell lines.

**Figure 2:**
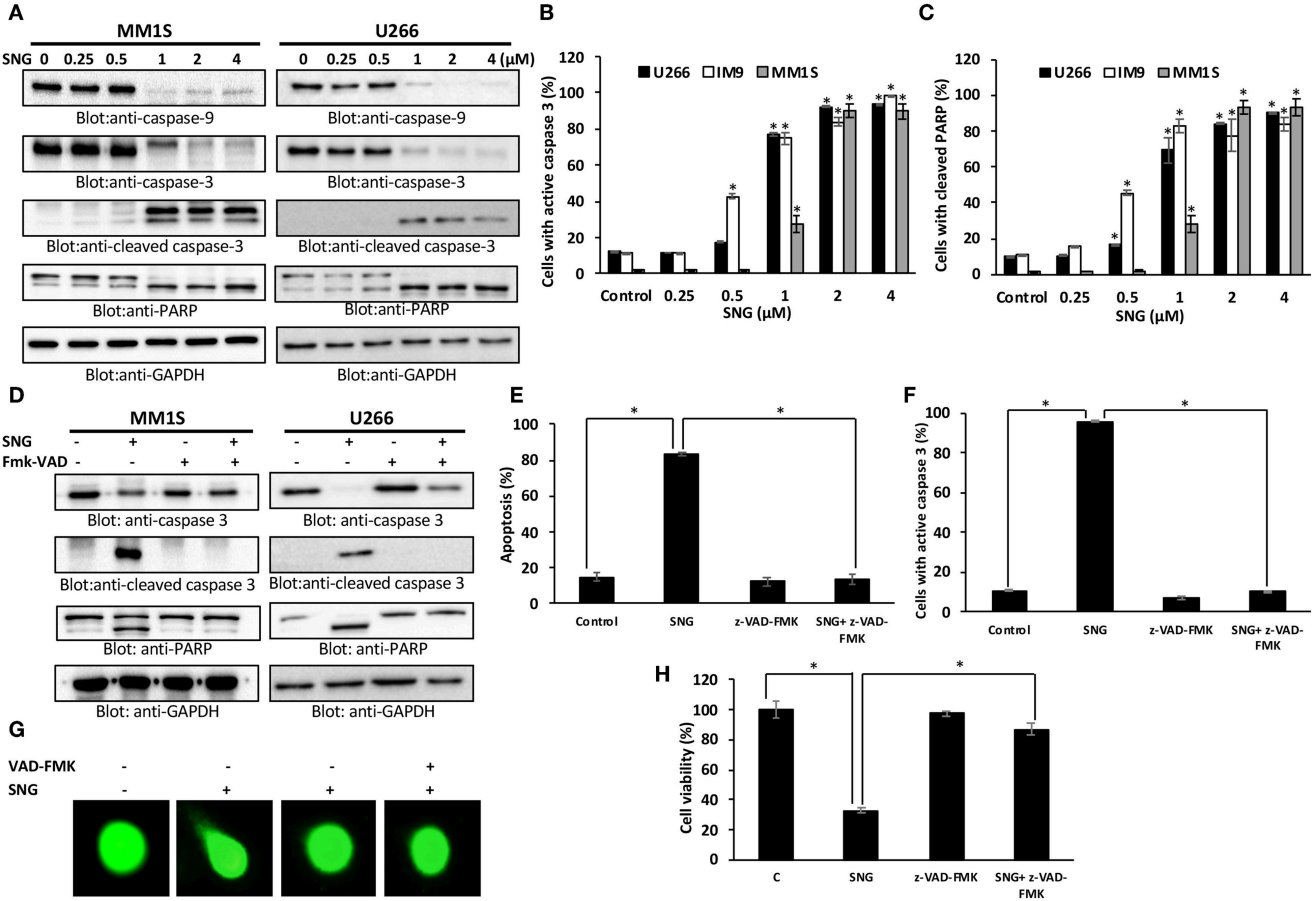
SNG mediated activation of caspase cascade in MM cells. SNG-induced activation of caspase-9, caspase-3, and cleavage of PARP in MM cells. **(A)** U266 and MM1S cells were treated with and without 0.25, 0.5, 1.0, 2.0, and 4 μM of SNG for 24 h. Cells were lysed and 50 μg of proteins were separated on SDS-PAGE, transferred to PVDF membrane, and immunoblotted with antibodies against caspase-9, caspase-3, cleaved caspase-3, PARP, and GAPDH. SNG–induced caspase-3 activation **(B)** and PARP cleavage **(C)** in MM cell lines. U266, IM9, and MM1S were treated with and without 0.25, 0.5, 1.0, 2.0, and 4 μM of SNG for 24 h and levels of active caspase-3 and cleaved PARP were determined by flow cytometry as described in Materials and Methods. The graph displays the mean ± SD (standard deviation). *p < 0.001. **(D)** Effect of z-VAD-FMK on SNG-induced caspase cascade. U266 and MM1S cells were pre-treated with 20 μM z-VAD-FMK for 1 h and subsequently treated with 2 μM SNG for 24 h. Cells were lysed and 50 μg of proteins were separated on SDS-PAGE, transferred to PVDF membrane, and immunoblotted with antibodies against caspase-3, cleaved caspase-3, PARP, and GAPDH. **(E)** Effect of z-VAD-FMK on SNG-induced apoptosis. U266 and MM1S cells were treated as described above and induction of apoptosis was measured using fluorescein-conjugated annexin-V and propidium iodide staining and analyzed by flow cytometry. The graph displays the mean ± SD (standard deviation) of the percentage of apoptotic cells (Early Apoptosis + Late Apoptosis). *p < 0.001**. (F)** Pre-treatment of z-VAD-FMK prevented SNG mediated caspase-3 activation and PARP cleavage. U266 and MM1S cells were treated as described above and analyzed for active caspase-3 and cleaved PARP by flow cytometry. The graph displays the mean ± SD (standard deviation). *p < 0.001. **(G)** Effect of z-VAD-FMK on SNG-induced DNA damage. U266 and MM1S cells were treated as described above and induction of DNA damage was visualized using Comet assay. **(H)** Effect of z-VAD-FMK on SNG-mediated inhibition of cell viability. U266 cells were pretreated with 20 μM Z-VAD-fmk for 1 h. Then cells were treated with 2 μM SNG 24 h and cell viability assay deterimed by CCK-8 kit as described in Methods. The graph displays the mean ± SD (standard deviation). *p < 0.001.

Caspases are proteolytic proteins that play an important role in the cell death signaling pathway for rapid and efficient apoptosis (47, 48). Our results showed that SNG treatment resulted in the activation of caspase-9 followed by the final executioner -caspase 3, leading to PARP cleavage in MM cells (Figure 2A). In addition, we also determined activation of caspase-3 and the subsequent PARP cleavage by flow cytometry to confirm the role of caspase cascade in SNG mediated induction of apoptosis. Treatment of MM cells with SNG up to 1 μM resulted in a dose-dependent increase in the cellular levels of active caspase 3 and cleaved PARP in all cell lines (Figures 2B,C) while reaching to a saturation level at 2 and 4 μM of SNG treatment condition. The involvement of caspase activation in SNG mediated apoptosis was further proved by the observation that pretreatment of MM cell with z- VAD-FMK, a universal inhibitor of caspases resulted in abrogation of SNG-induced apoptosis, caspase signaling, DNA damage, and cell viability (Figures 2D–H and Supplementary Figures 2A–C). All of the above-discussed data substantiate the role of caspase-mediated signaling pathways in SNG-induced apoptosis in MM cells.

### SNG Activates the Intrinsic Apoptotic Pathway in Multiple Myeloma Cells

**Figure 3:**
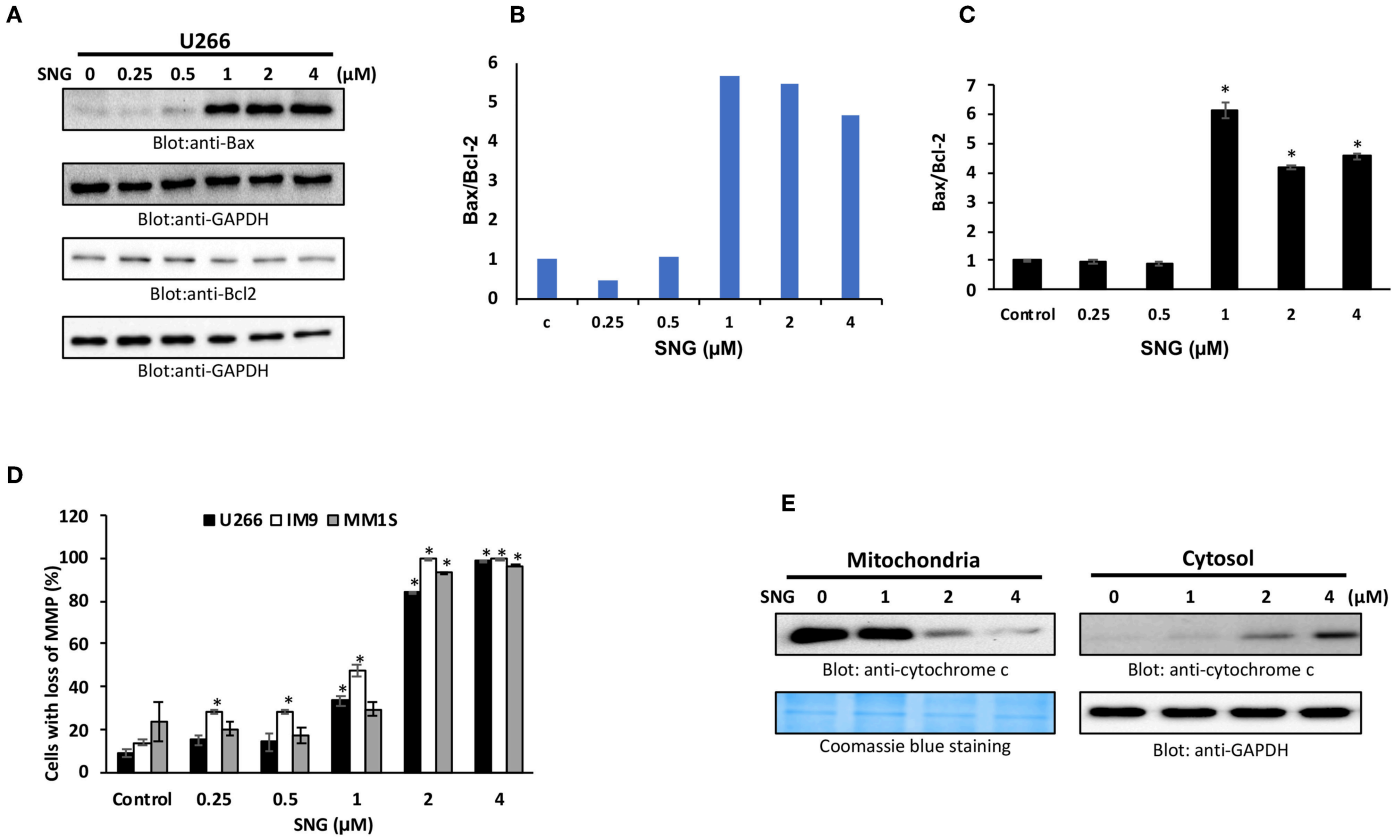
SNG-induced mitochondrial signaling pathways in MM cells. SNG treatment causes an alteration in Bcl-2 expression. **(A)** U266 cells were treated with increasing doses of SNG for 24 h as indicated. After cell lysis, equal amounts of proteins were separated by SDS–PAGE, transferred to PVDF membrane and immunoblotted with antibodies against Bax, Bcl-2, and GAPDH as indicated. **(B)** Data obtained from immunoblot analyses of Bax and Bcl-2. U266 cells were used to evaluate effects of SNG on Bax/Bcl-2 ratio. Densitometric analysis of Bax and Bcl-two bands was performed using AlphaImager Software (San Leandro, CA, USA), and data (relative density normalized to GAPDH) were plotted as Bax/Bcl-2 ratio. **(C)** U266 cells were treated with and without 0.25, 0.5, 1.0, 2.0, and 4 μM of SNG for 24 h and levels of Bax and Bcl-2 were determined by flow cytometry as described in Materials and Methods. The MFI values were used to calculate the Bax/Bcl-2 ratio, and the mean ± SD (standard deviation) is plotted in the graph. * *p* < 0.001. **(D)** SNG treatment causes the loss of MMP in MM cells. U266, IM9, and MM1S cells were treated with indicated doses of SNG for 24 h. After JC1 staining, cells were analyzed by flow cytometry as described in Materials and Methods. The graph displays the mean ± SD of three independent experiments. *p < 0.001. **(E)** The SNG-induced release of cytochrome c. U266 cells were treated with and without 1.0, 2.0, and 4.0 μM SNG for 24 h. Cytoplasmic fraction was isolated as described in Materials and Methods. Cell extracts were separated on SDS-PAGE, transferred to PVDF membrane, and immunoblotted with an antibody against cytochrome c and GAPDH.

Members of the Bcl-2 family is known to regulate apoptosis by keeping a balance between the levels of anti-apoptotic genes, such as Bcl-2, Bclxl, and the pro-apoptotic molecules with BH3-domain (Bax, Bak, etc.). Any deviation from this delicate balance can lead to activation or inhibition of mitochondria-mediated death pathway (49). Therefore, we sought to evaluate the effect of SNG on the protein levels of the most important members of the Bcl-2 family; Bax and Bcl-2, in U266 cells. As shown in Figures 3A–C, SNG treatment resulted in a significant decrease in Bcl-2 expression in a dose-dependent manner which coincides with the increase in Bax. Densitometric analysis of the blots and flow cytometry analysis of Bax and Bcl2 proteins confirms the rise in the ratio of Bax to Bcl-2 protein levels (Figures 3A–C), which is known to occur during mitochondria-mediated apoptosis (50).

The permeability of the mitochondrial outer membrane, as well as the proton gradient across the inner membrane, can be affected by alterations in the Bax/Bcl2 ratio, which can lead to the loss of membrane potential. So, we designed further experiments to study MMP as well as the cytosolic release of cytochrome c (normally sequestered within the mitochondria) in various MM cell lines with and without SNG treatment. After SNG treatment, the transmembrane potential in MM cells was analyzed using JC1 dye by flow cytometry as depolarization is associated with early stages of the apoptotic process. As shown in Figure 3D, there was a significant (*p* < 0.001) depolarization of the membrane in a dose-dependent manner in all the MM cell lines. SNG treatment resulted in an increase in the cytoplasmic level of cytochrome c with a concomitant decrease in mitochondrial fractions (Figure 3E). Cytosolic cytochrome c triggers downstream events in the apoptotic cascade, including the activation of caspase cascade which is a hallmark marker of programmed cell death (51).

### SNG Promotes Oxidative Stress via ROS Generation in Multiple Myeloma Cells

**Figure 4:**
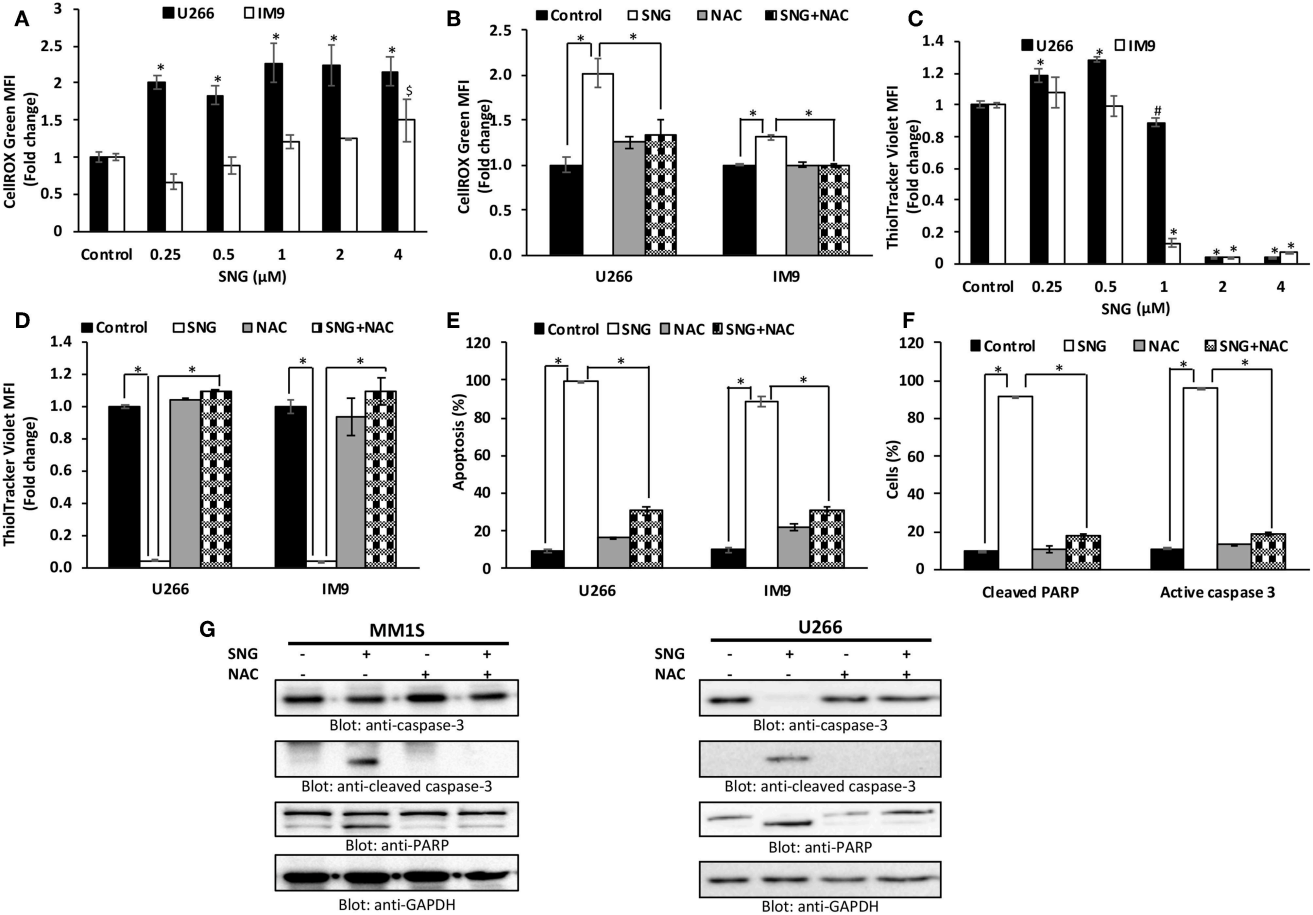
SNG increases ROS generation in MM cells. **(A)** U266 and IM9 cells were treated with SNG for 24 h, and the cellular level of ROS was measured using CellROX Green reagent by flow cytometry as described in Methods. The graph displays the mean ± SD (standard deviation) of ROS levels (fold change compared to control) of three experiments *p < 0.001. **(B)** Effect of NAC on SNG-induced generation of ROS. U266 and IM9 cells were pre-treated with 10 mM NAC, subsequently treated with 2 μM SNG for 24 h. CellROX assays were performed as described in Methods. The graph displays the mean ± SD (standard deviation) of ROS levels (fold change compared to control) of three experiments *p < 0.001. **(C)** Effect of SNG on reduced glutathione levels in MM cell lines. U266 and IM9 cells were treated with increasing doses of SNG for 24 h and levels of reduced glutathione were determined using ThiolTracker Violet reagent by flow cytometry. The graph displays the mean ± SD (standard deviation) of ThiolTracker Violet MFI (fold change compared to control) of three experiments. ^#^*p* < 0.05, *p < 0.001. **(D)** NAC pre-treatment of MM cell prevents SNG-induced glutathione depletion. U266 and IM9 cells were pretreated with 10 mM NAC, subsequently treated with 2 μM SNG as indicated for 24 h and GSH content was determined by ThiolTracker Violet by flow cytometry. Bars represent mean ± SD (standard deviation), *p < 0.001. **(E)** Pre-treatment of MM cell with NAC prevent SNG-induced apoptosis. U266 and IM9 cells were pretreated with 10 mM NAC, subsequently treated with 2 μM SNG as indicated for 24 h. The cells were then stained with fluorescein-conjugated annexin-V, PI, and analyzed by flow cytometry. The graph displays the mean ± SD (standard deviation) of the percentage of apoptotic cells (Early Apoptosis + Late Apoptosis). *P < 0.001. **(F)** Pre-treatment of MM cell with NAC prevent SNG induced PARP cleavage and Caspase-3 activity. U266 cells were pretreated with 10 mM NAC, subsequently treated with 2 μM SNG as indicated for 24 h and levels of active caspase-3 and cleaved PARP were determined by flow cytometry as described in Materials and Methods. The graph displays the mean ± SD (standard deviation) *p < 0.001. **(G)** Effect of NAC on SNG-induced caspase cascade. U266 and MM1S cells were pre-treated with 10 mM NAC and subsequently treated with 2 μM SNG for 24 h. Cells were lysed and 50 μg of proteins were separated on SDS-PAGE, transferred to PVDF membrane, and immunoblotted with antibodies against caspase-3, cleaved caspase-3, PARP, and GAPDH.

Oxidative stress results from an imbalance between ROS production and the cells ability to scavenge them (52). ROS are a group of highly reactive molecules responsible for regulating normal cell proliferation and differentiation (53). Under normal physiological conditions, ROS function as “redox messengers” in low levels mediating the growth adaptation and overall survival of the cell (54). However, the high level of these free radical molecules mediates cellular DNA damage, reduces normal MMP, and promotes cancer cell death. SNG has been shown to act as a ROS inducer via increasing ROS-related stress resulting in ROS-dependent apoptosis in cancer cells (16). Thus, we had an interest in identifying if SNG could induce ROS-dependent apoptosis specifically in MM cells. Therefore, we measured oxidative stress in SNG treated U266 and IM9 cells using the fluorogenic probe CellROX Green which emits a strong fluorescence (peak emission at 525 nm) when oxidized by ROS. U266 and IM9 cells were treated with SNG for 24 h at various concentrations. SNG treated MM cells showed a dose-dependent generation of ROS at the cellular level (Figure 4A). Pretreatment of MM cell with N-acetyl-L-cysteine (NAC) an inhibitor of ROS generation abolished significantly SNG-mediated ROS in both cell lines (Figure 4B). Glutathione (GSH) is key component of cellular antioxidant defense mechanism and has a vital role in many oxidative stress-related diseases, including cancer. In the next experiment, we evaluated whether SNG treatment of MM cells reduced GSH level in MM cells. As shown in Figure 4C, SNG treatment of U266 and IM9 resulted in a dose-dependent depletion of GSH which was statistically significant. Furthermore, when the MM cells were pretreated with 10 mM NAC, the SNG mediated deletion of GSH in MM cells was prevented (Figure 4D). We determined in the next series of experiments whether SNG mediated generation of ROS is involved in the activation of caspase-mediated apoptosis in MM cells. U266 and IM9 cells were initially incubated with 10 mM NAC, followed by SNG treatment for 24 h. As shown in Figures 4E–G, pretreatment of MM cells with NAC prevented apoptosis as well as caspase activity strongly indicating that SNG mediated ROS is involved in the induction of apoptosis in MM cells.

### SNG Suppresses JAK2/STAT3 Survival Pathway in Multiple Myeloma Cells

**Figure 5:**
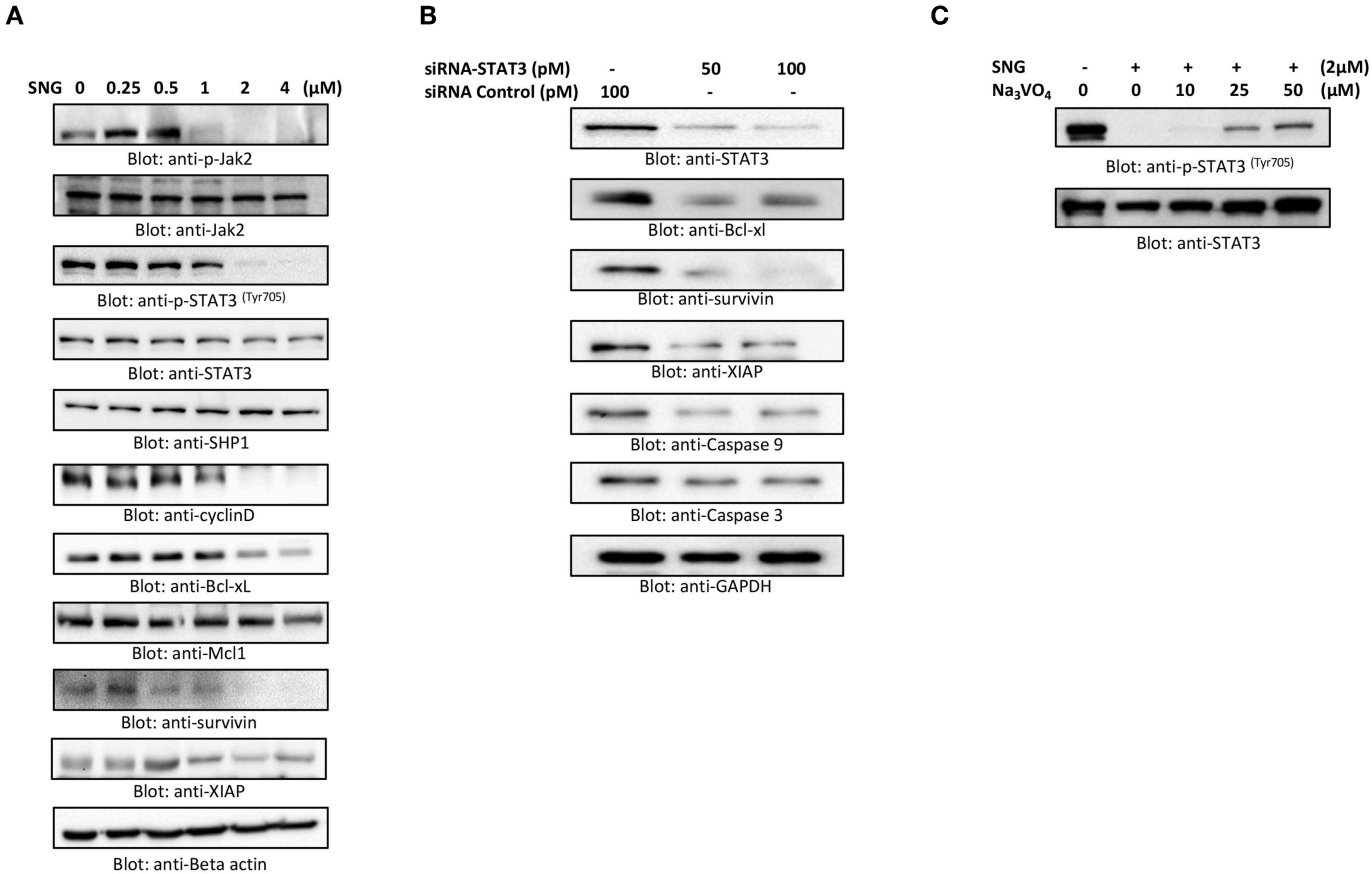
SNG suppresses constitutively activated STAT3 signaling pathway. **(A)** U266 cells were treated with increasing doses of SNG for 24 h as indicated. After cell lysis, equal amounts of proteins were separated by SDS–PAGE, transferred to PVDF membrane, and immunoblotted with antibodies of p-JAK2, JAK2, p-STAT3 ^(Try705)^, STAT3, SHP-1, Cyclin D, Bclxl, Mcl1, survivin, XIAP, and GAPDH. **(B)** Effect of Stat3 knock-down on total STAT3 levels and related proteins. U266 cells were transfected with either control (100 pM) or STAT3 specific siRNA (50 or 100 pM). Cells extracts were separated on SDS-PAGE, transferred to PVDF membrane, and immunoblotted with antibodies against STAT3, Bclxl, survivin, XIAP, caspase-3, and caspase-9. **(C)** Involvement of Tyrosine-protein phosphatase in SNG induced reduction of Stat3 phosphorylation. U266 cells were treated with 2.0 μM SNG in the absence or presence of various doses of sodium pervanadate for 4 h, cell extracts were prepared, and Western blotting was performed using p-STAT3 and STAT3 as indicated.

STAT3 is known to mediate cell survival in many cancers, and U266 cell line is reported to have constitutively activated STAT3 pathway. So, we analyzed the effect of SNG on the phosphorylation and activation of STAT3 in MM cells by treating the cells with increasing concentrations of SNG for 24 h. SNG treatment resulted in the dephosphorylation of STAT3, which was completely abolished at concentrations 2 and 4 μM (Figure 5A). Then, we checked the effect of SNG on the total protein levels of STAT3. As shown in Figure 5A SNG treatment did not show any effects on total STAT3 proteins. STAT3 has been shown to be regulated by no-receptor tyrosine kinase JAK2 (55). Thus, we sought to find out whether SNG mediated suppression of STAT3 is JAK2 dependent. As shown in Figure 5A, SNG treatment of U266 cell causes dephosphorylation of JAK2 without affecting its protein level. Interestingly, SNG did not show any effects on the phosphorylation of JAK1 as well as on p-STAT3-ser727 in U266 cells (Supplementary Figure 3). To further characterize the functional effects of SNG-mediated inhibition of STAT3 activity (dephosphorylation), we investigated the STAT3 downstream target gene products including cyclin D, Bclxl, and XIAP that are involved in proliferation and growth of MM cells. As shown in Figure 5A, SNG treatment of MM cells resulted in decreased expression of cyclin D, with maximal effect observed at the doses 2 and 4 μM. Further, we studied the involvement of other antiapoptotic proteins in SNG-induced apoptosis. X-linked Inhibitor of Apoptosis protein (XIAP) over-expression (56, 57) has been reported to correlate with the suppression of apoptosis in many cancer cells (58), and it is the most promising target of Inhibitor of Apoptosis proteins (IAPs) family which is also selectively over-expressed in various cancers including MM (59–62). In diagnosed MM patients, XIAP expression level is high and decreased XIAP expression correlates with the treatment efficacy of chemotherapy and proteasome inhibitors (63). Therefore, we determined the effect of SNG on MM cell lines. As shown in Figure 5A, U266 cells had higher levels of XIAP in the control as expected, but a dose-dependent decrease in XIAP expression was seen upon treatment with SNG. To provide direct evidence, whether STAT3 down-regulates antiapoptotic proteins such as Bclxl, survivin and XIAP in U266 cells, we used RNA interference to knock down STAT3 using siRNA against STAT3. As shown in Figure 5B, STAT3-specific siRNA decreased the protein levels of STAT3, which lead to down-regulation of Bclxl, survivin, and XIAP, in the U266 cells. In addition, gene silencing of STAT3 resulted in activation of the caspases, −3 and −9 (Figure 5B). These results suggest that targeting STAT3 signaling either with SNG treatment or gene silencing with siRNA resulted in downregulation of antiapoptotic genes and activation of caspases that lead to induction of apoptosis in MM cells.

PTPs are negative regulators of the JAK/STAT signaling (64). Therefore, we sought to determine whether SNG-induced de-phosphorylation (inactivation) of STAT3 is involving PTPs. As shown in Figure 5A SNG treatment upregulates the expression of SHP-1 in a dose-dependent manner. Moreover, pretreatment of U266 cells with sodium pervanadate, a PTP inhibitor prevented SNG-mediated dephosphorylation (inactivation) of STAT3 (Figure 5C), suggesting the role of SHP-1 in SNG-mediated STAT3 de-phosphorylation (inactivation).

### SNG Treatment of MM Cells Prevents IL 6 Secretion and Inhibits IL6 Inducible STAT3 Activation

**Figure 6:**
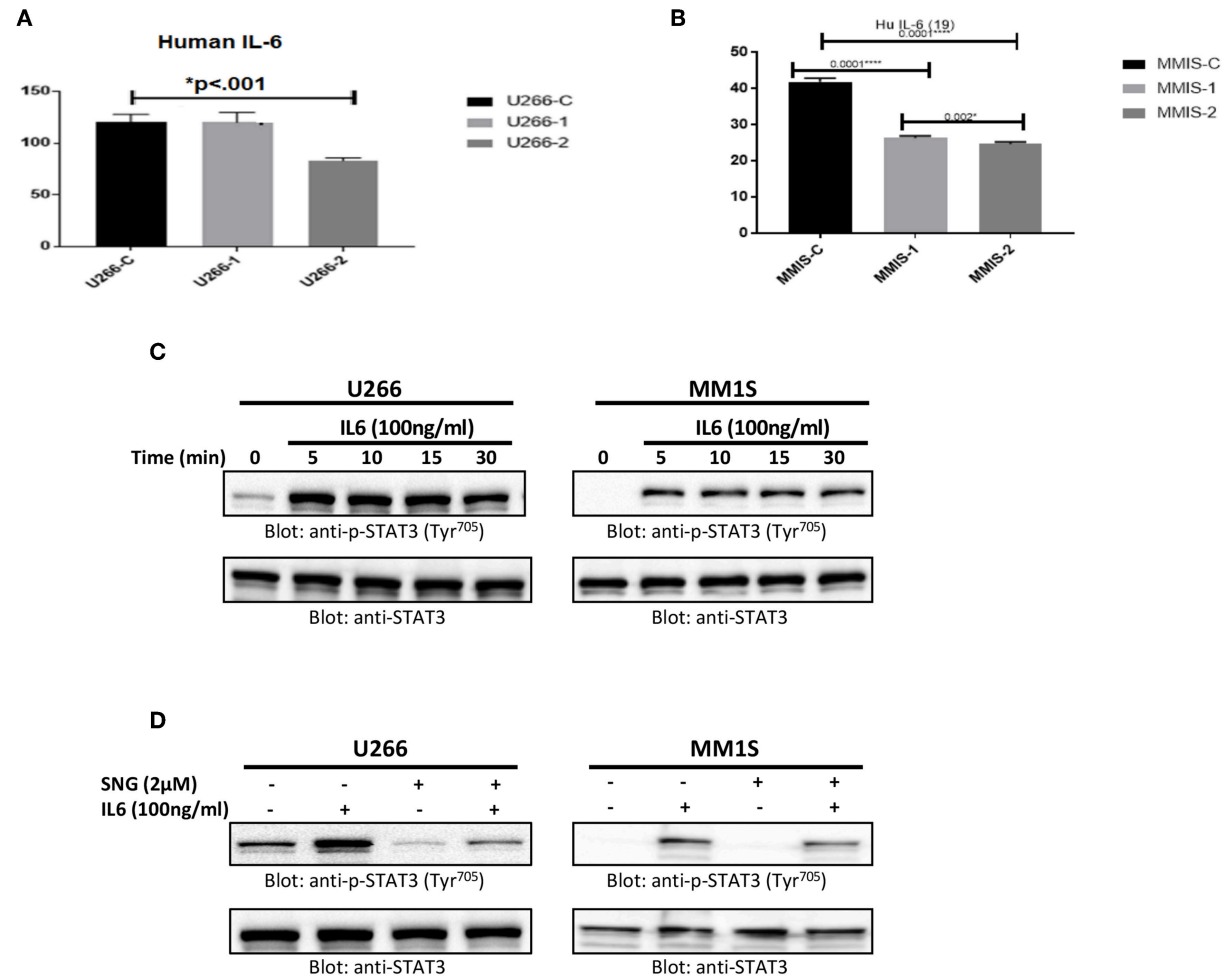
SNG mediated inhibition of IL 6 secretion and IL 6 induced STAT3 activation in MM cells. U266 **(A)** and MM1S **(B)** cells were treated with increasing doses of SNG for 24 h as indicated. The culture supernatant was used to estimate the levels of IL 6 (pg/ml) as described by cytokine measement kit. The graph displays the mean ± SD (standard deviation) of three independent of for all the doses. *p < 0.001 **(C)** U266 and MM1S cells were serum satrved for 24 h and subsequently treated with IL6 (100 ng/ml) for indicated time periods. After cell lysis, equal amounts of proteins were separated by SDS–PAGE, transferred to PVDF membrane and immunoblotted with antibodies of p-STAT3 ^(Try705)^ and STAT3. **(D)** SNG inhibits IL6 induced STAT3 activation. U266 and MM1S cells were serum sartved for 24 h, subsequently pretreated with 2.0 μM SNG for 1 h and then stimulated with IL 6 (100 μg/ml) for 30 min. Cell extracts were prepared, and Western blotting was performed using antibodies against p-STAT3 and STAT3 as described earlier.

U266 and MM1S cells were treated with 1 and 2 μM of SNG for 24 h. Secretion of IL6 in cell culture media was measured by a Multiplexing kit as described in material and methods. As shown in Figure 6A, the secretion level of IL6 is comparatively higher in U266 as compared to MM1S confirming earlier findings by Lin et al. (65). Interestingly, SNG treatment of MM cells prevented the secretion of IL6 secretion into the media (Figures 6A,B). IL-6 has been shown to activate STAT3. Hence, we checked whether IL6 could induce phosphorylation of STAT3 of MM cells. U266 and MM1S cells were treated with 100 ng/ml of IL6 for various time-periods. Treatment of MM cells with IL6 causes time-dependent phosphorylation of STAT3 in both cell lines (Figure 6C). Thymoquinone has been shown to prevent IL-6 inducible p-SAT3 in MM cells (66), we also analyzed whether SNG can prevent IL6 mediated-STAT3 activation. As shown in Figure 6D, pretreatment of MM cell with SNG prevented IL6-mediated STAT3 phosphorylation suggesting that SNG Inhibits IL6 inducible STAT3 activation (Figure 6D).

### Combination of SNG and Bortezomib Enhances Cytotoxicity in Multiple Myeloma Cells

**Figure 7:**
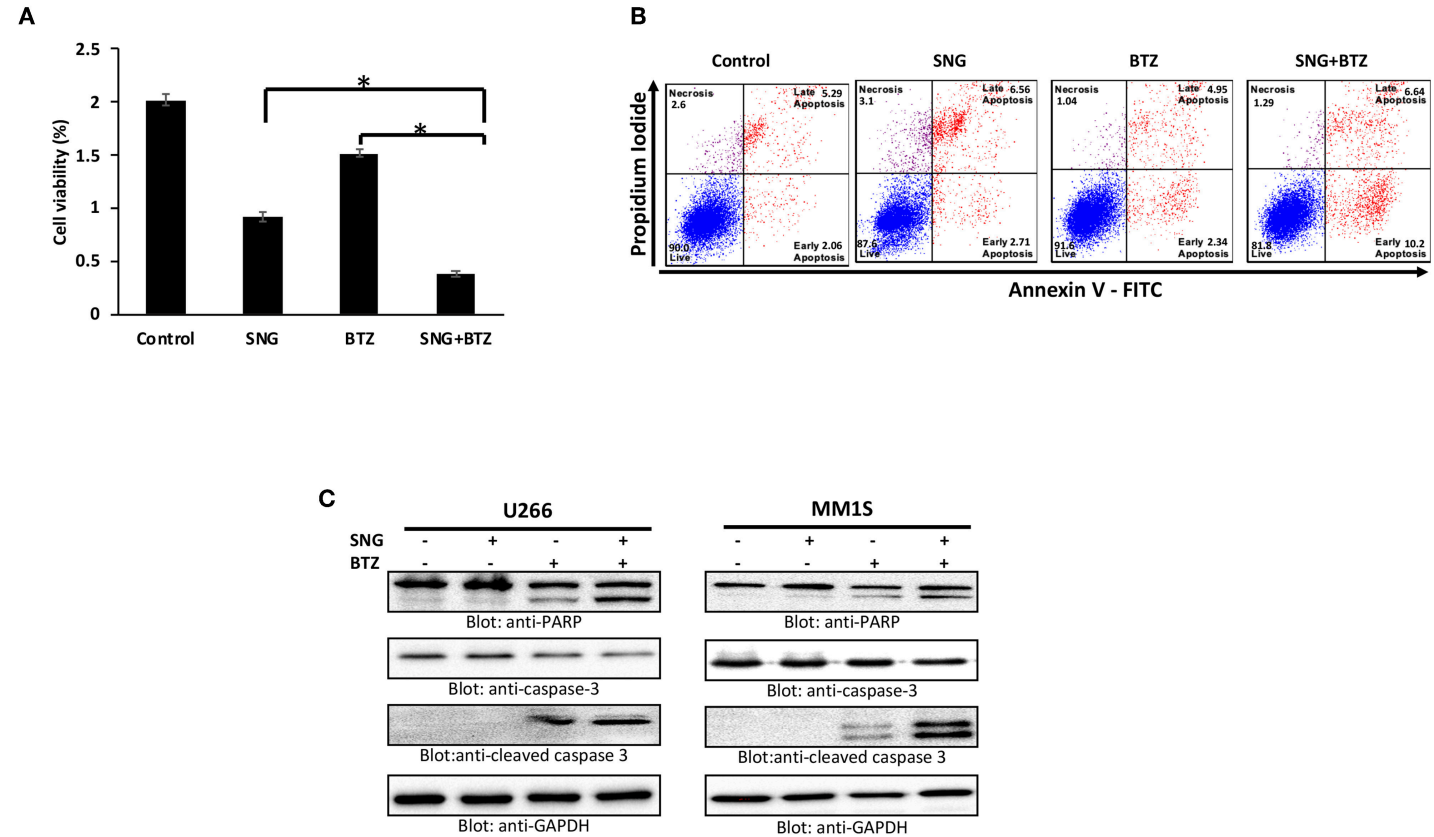
SNG enhances the cytotoxicity of bortezomib in MM cells. **(A)** U266 cells were treated with 0.5 μM SNG and 5 nM BTZ alone or in combination for 24 h as indicated. Cell proliferation assays were performed using CCK-8 as described in Materials and Methods. The graph displays the mean ± SD (standard deviation) of three independent experiments with replicates of six wells for all the doses. *p < 0.001. **(B)** U266 cells were treated with SNG and BTZ as indicated earlier. The cells were then stained with fluorescein-conjugated annexin-V, PI, and analyzed by flow cytometry. **(C)** U266 cells were treated with SNG and BTZ as indicated earlier. Cells were lysed and 50 μg of proteins were separated on SDS-PAGE, transferred to PVDF membrane, and immunoblotted with antibodies against caspase-3, cleaved caspase-3, PARP, and GAPDH.

Bortezomib (BTZ) is a selective, first-in-class, FDA-approved proteasome inhibitor for the treatment of MM patients (43). However, the efficacy of BTZ is mostly constrained due to the development of resistance (67) and relapse (68) which are wide-spread. Many natural compounds have been shown to potentiated the anticancer activity of bortezomib *in vitro* as well *in vivo* (69, 70) therefore, we sought to determine whether SNG can increase the anticancer effects of BTZ in MM cells when used as a combination treatment. To this end, we treated U266 cells with sub toxic doses of SNG (0.5 μM) and BTZ (5 nM) alone or in a combination of SNG and BTZ. As shown in Figure 7, the combination of SNG and BTZ reduced cell proliferation significantly (Figure 7A) as compared to SNG or BTZ single treatment condition. In addition, this combination dose was able to enhance cell death via activation of caspase−3 and PARP (Figures 7B,C).

## Discussion

Previous studies showed that Sanguinarine (SNG) prevented carcinogenic activity in various types of cancer cell lines. The major mechanisms of anti-cancer activity described for SNG was its role in triggering intrinsic apoptosis, stimulating the generation of reactive oxygen species (ROS), and inducing DNA fragmentation (16, 71–73). However, SNG activity was not yet defined in Multiple myeloma (MM). Multiple myeloma is a slow proliferative B cell malignancy that leads to a buildup of apoptosis-resistant plasma cells in the bone marrow (1, 74). In the current study, we investigated the anticancer effect of SNG in MM cells. We performed a panel of tests to define the mechanism of activity of SNG in four MM cancer cell lines. Our findings strongly showed that SNG suppressed MM cells proliferation through the generation of apoptotic effects. The evidence for proapoptotic activity of SNG was revealed by increased annexin-V/PI positive cells, activation of caspase-3 and cleavage of PARP in MM cells after SNG treatment. It is well-known that the balance of pro-apoptotic (Bax) and anti-apoptotic (Bcl-2) proteins of the Bcl-2 family, plays a role in the integrity of the mitochondrial membrane (75). Bax has been shown to antagonize Bcl-2 expression leading to an alteration of the mitochondrial membrane permeability followed by activation of caspases and apoptosis (76). We are showing that SNG treatment enhanced the Bax/Bcl-2 ratio in a dose-dependent manner. Indeed, SNG treatment induces an alteration of Bcl-2 expression and enhancement of Bax levels resulting in a significant alteration in mitochondrial membrane potential (MMP). Consequently, we observed that treated MM cells presented an increase in cytochrome c levels in their cytosolic fraction and a concomitant loss in their mitochondrial fraction. One of the functions of cytosolic cytochrome C is to interact with apoptotic protease-activating factor-1 (APAF-1) and procaspase 9 in the presence of ATP to form the multimeric complex, apoptosome, which in turn activates caspase 9 and induce apoptosis (34). Since we show that caspase 9 expression was efficiently increased in MM cells in response to SNG treatment, we suggest that SNG-mediated apoptosis in MM cell lines involves mitochondria-mediated caspase activity. Moreover, SNG caspase-dependent apoptotic effect was again confirmed by using z-VAD-FMK, a universal inhibitor of caspases, which prevented SNG-mediated apoptosis.

ROS generation and resulting oxidative stress are associated with cell death in many types of cells (30). In the present study, we showed that SNG treatment significantly induced the production of ROS along with a depletion of glutathione (GSH) level in MM cells. Furthermore, pretreatment of MM cells with N-acetylcysteine (NAC) seems to inhibit SNG-induced proapoptotic effects including depletion of GSH, apoptosis and activation of caspases and PARP. These findings strongly suggest that SNG mediated proapoptotic effects in MM cells occurs through the involvement of ROS.

Recent studies showed that deregulated intracellular signaling of the JAK/STAT3 pathway plays a role in the emergence of resistance to apoptosis in many human malignancies (77). Indeed, constitutive JAK/STAT and NF-κB signaling have been linked with the pathogenesis of MM (78, 79) and activated STAT3 was shown to control the transcription of many antiapoptotic genes such as Bcl2, survivin, and Bclxl (80, 81). Upregulation of these antiapoptotic proteins causes sustained cell survival, enhances cellular resistance to chemotherapy and suppresses apoptosis (82). Our data showed that SNG treatment leads to suppression of constitutive phosphorylation of STAT3 in MM cells. In addition, we also demonstrated that SNG inhibited the phosphorylation of JAK2, an upstream tyrosine kinase that regulates the activation of STAT3 (83). Furthermore, we observed that U266 cells treated with SNG had a downregulated expression of cyclin D, a proliferative marker of MM cells. SNG also suppressed the expression of antiapoptotic genes; survivin, Bclxl, and Bcl2, in a dose-dependent manner. Interestingly, gene knockout of STAT3 in U266 cells using specific STAT3 siRNA abolished the expression of survivin, Bclxl, and XIAP as well as activated caspase-9 and caspase-3.

Moreover, we investigated the effect of SNG on the SHP-1 molecule, a Protein tyrosine phosphatases (PTPs) which would regulate the activation of STAT3 (84). Our data showed that SNG treatment upregulated SHP-1 and this effect was concomitant with a decrease in phosphorylation of STAT3 in MM cells. To further confirm the role of SHP-1 in STAT3 activation/phosphorylation, we used pervanadate, an inhibitor of PTPs. In the presence of pervanadate, SNG treatment failed to suppress the phosphorylation of STAT3. Finally, our data shows for the first time, that SNG inhibits secretion of IL6 by MM cells, and suppresses IL-6 mediated signaling pathway in U266 cells. Interestingly, SNG also potentiated the anticancer effects of bortezomib (BTZ) in MM cells suggesting that SNG may be a novel therapeutic agent for management of MM treatment alone or in combination with other anticancer agents.

**Figure 8:**
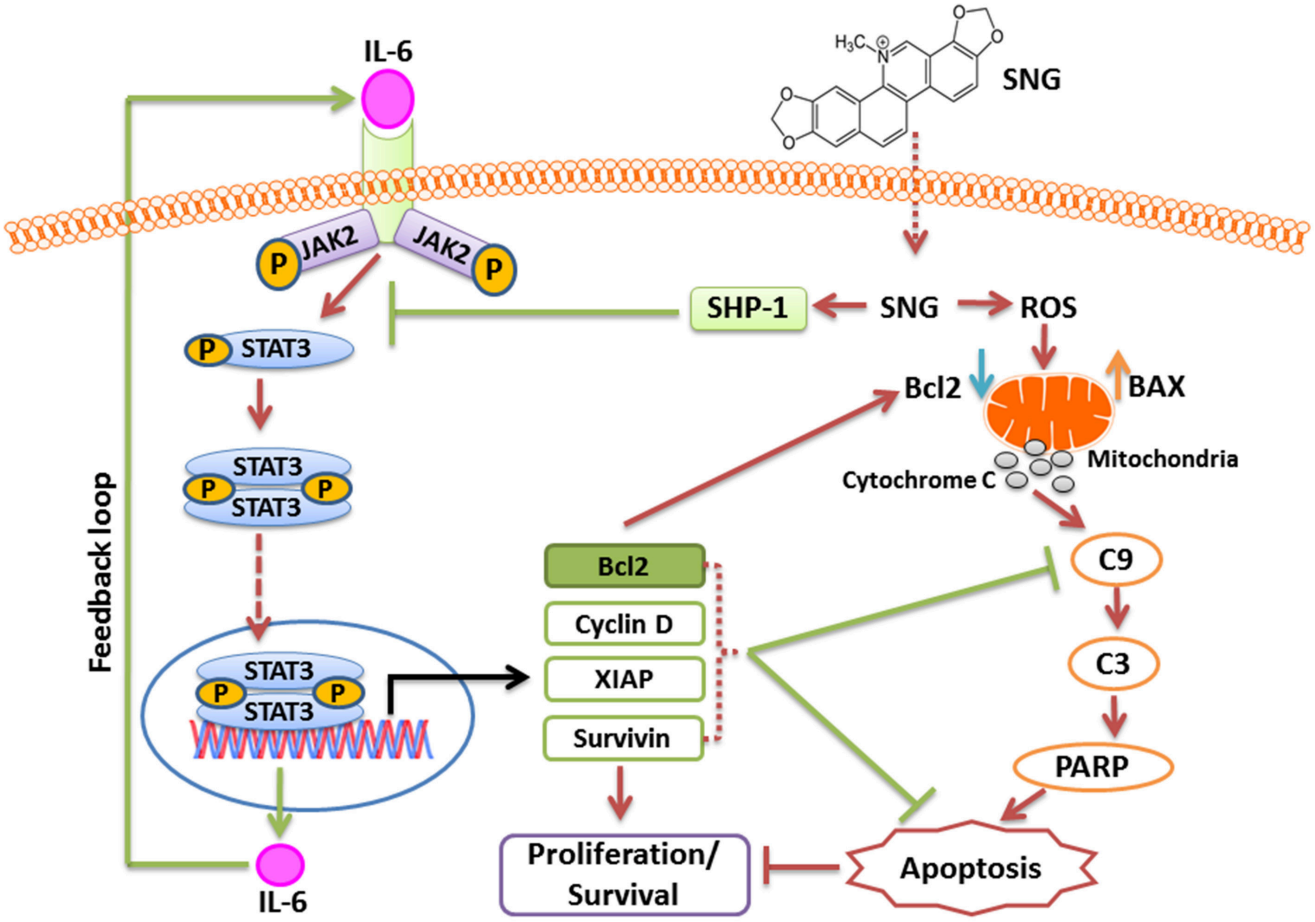
Schematic diagram of SNG mediated action in MM cells.

In summary, our study is the first one to define the mechanism of activity of SNG in MM cells. We obtained significant data that provides evidence about the cytotoxic effect of SNG in this malignancy. Indeed, we showed that SNG treatment triggered cytotoxic effects by inactivation of JAK/STAT3 activity via suppression of anti-apoptotic genes (schematically depicted in Figure 8). We have described a novel mechanism of action of SNG by showing that it upregulates SHP-1 protein and consequently inhibit phosphorylation and activation of STAT3. In addition, we showed that SNG caused activation of intrinsic apoptotic pathways in MM cells and these findings are in concordance with previous studies that described the same mechanism of activity but in different types of tumor cells. Moreover, we demonstrated that SNG induces anticancer activity in MM cells by depletion of glutathione resulting in the generation of ROS.

Most importantly, we observed that anti-cancer activity of the chemotherapeutic drug bortezomib was enhanced when combined with SNG treatment in MM cells. These findings provide fundamental information for the design and selection of combinatory therapeutic approach to treat MM. The main mechanism of action described for bortezomib in MM is proteasome inhibition which results in endoplasmic reticulum stress-induced apoptosis, accumulation of ROS, and deregulation of functional proteins (such as IL-6, TNF-α, NF-κB) (85). We think that it would be interesting to investigate the effect of SNG on some additional functional protein such as NF-κB to understand how it can potentiate the anti-cancer activity of BTZ. Currently, our objective is to perform further studies in MM animal models and in MM human samples for confirmation of our findings and for a better understanding *in vivo* of the link between the different signaling pathways affected by SNG treatment.

## Author Contributions

SA, IA, and KS performed experiment and help in experiment designing, data analysis, and manuscript writing. SK, KP, AK, EA, FS, JJ, AR, and MM performed experiment and data analysis. HE, RT, HO, HZ, SD, and MS help manuscript writing, editing, proofreading. SU experiment design, data analysis and manuscript writing, and proofreading.

### Conflict of Interest Statement

The authors declare that the research was conducted in the absence of any commercial or financial relationships that could be construed as a potential conflict of interest.
