# Supplementary figures and images for: Sanguinarine Induces Apoptosis Pathway in Multiple Myeloma Cell Lines via Inhibition of the JaK2/STAT3 Signaling
Source: Front Oncol. 2019 Apr 17;9:285. doi: 10.3389/fonc.2019.00285 (PMC6478801; doi:10.3389/fonc.2019.00285)

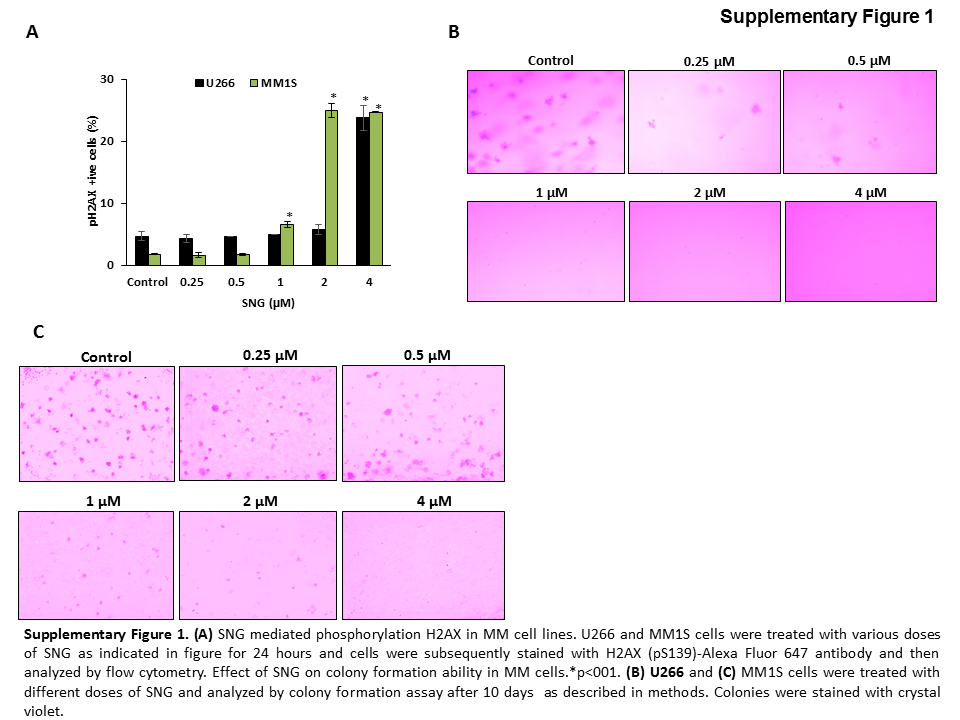

Supplement: Supplementary file 1 [file Image_1.tif]

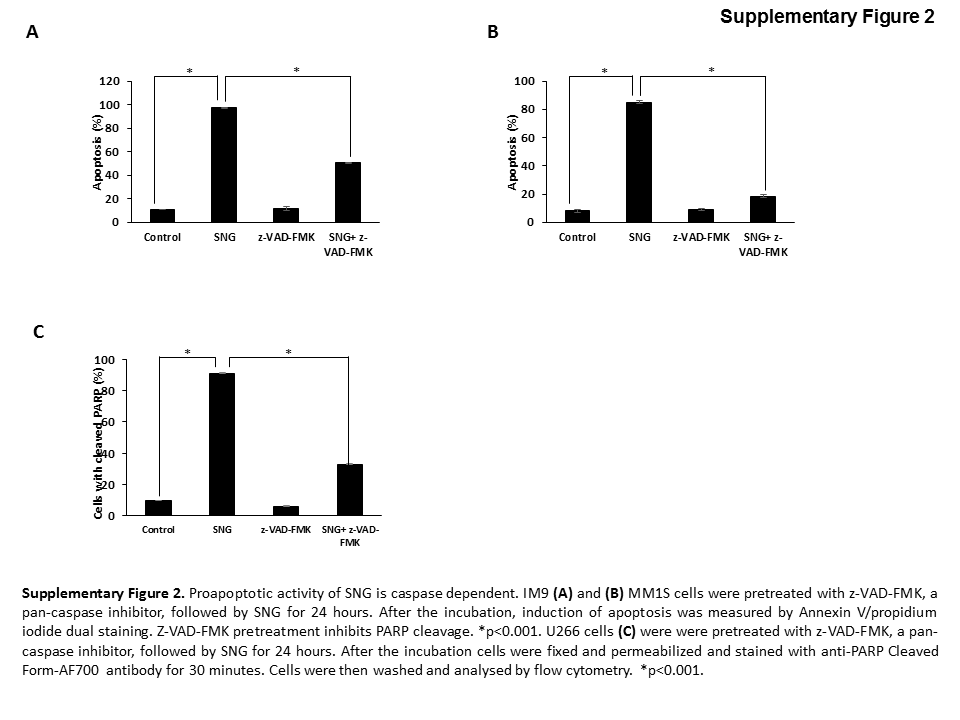

Supplement: Supplementary file 2 [file Image_2.tif]

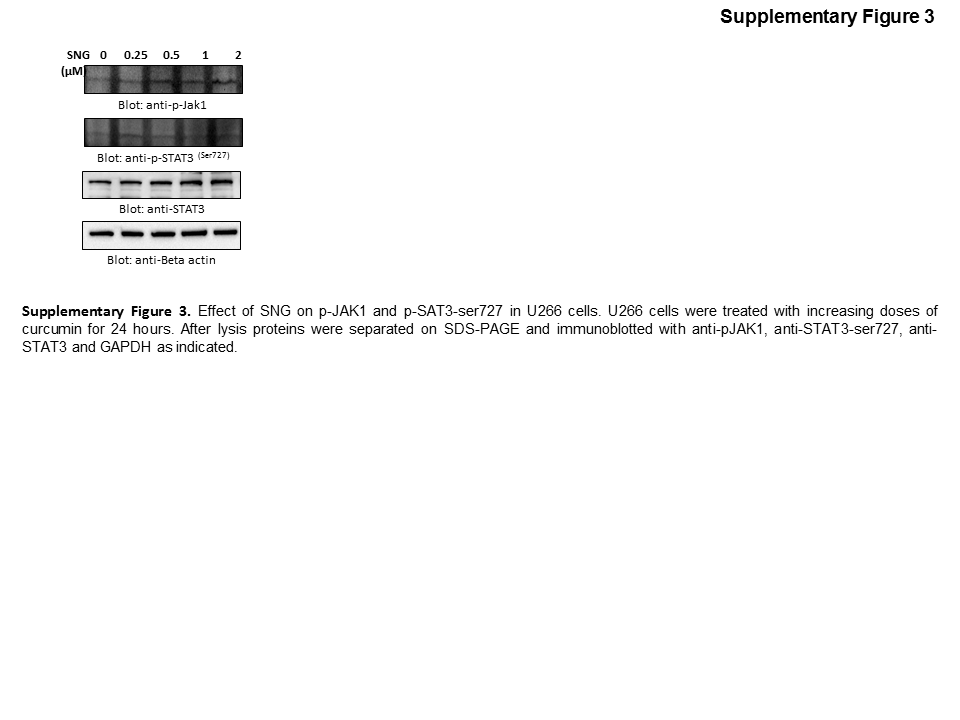

Supplement: Supplementary file 3 [file Image_3.tif]
